# Web-based versus simulation-based refresher training in newborn life support: A randomized multicenter noninferiority controlled trial

**DOI:** 10.1016/j.resplu.2026.101382

**Published:** 2026-06-10

**Authors:** Kathleen Parthey, Anne Goldhahn, Anselm Linke, Katja Raberger, A. Wienke, Dietrich Stoevesandt, Roland Haase, for the NeoLITE-Study

**Affiliations:** a Department of Pediatrics II, University Hospital, Martin Luther University Halle-Wittenberg, Halle, Germany; b University Medicine Halle, Dorothea-Erxleben Learning Center Halle (DELH), Germany; c Department of Pediatrics I, University Hospital, Martin Luther University Halle-Wittenberg, Halle, Germany; d University Medicine Halle, Institute for Medical Epidemiology, Biometrics and Informatics, Germany

**Keywords:** Newborn, Life support, Training, Simulation, Web-based, Neonatal

## Abstract

**Background:**

Regular training for healthcare professionals in newborn life support is life-saving. Web-based refresher has the potential to be non-inferior to simulation-based refresher training.

**Methods:**

The multicenter non-inferiority randomized controlled trial was conducted in six hospitals Level I–IV in Germany. Following an initial full-scale, high-fidelity simulation training, we compared a web-based refresher with a simulation-based refresher. The professionals were assessed at three points in time, using a pseudonymised questionnaire and a Megacode test, scores added up to a sum score. Assessment was performed prior to, and immediately after the initial training and three months after the intervention. The primary aim was to analyze whether a web-based refresher is non-inferior in avoiding skill decay. Participation at the refresher and recurring errors were secondary outcomes.

**Results:**

Of 228 randomized healthcare professionals, 139 (web-based refresher group *n* = 78 vs. simulation-based refresher group *n* = 61) completed the study per protocol. The mean sum scores ± SD 3 months after the refresher was 47.0 ± 5.6 in the simulation-based group and 45.2 ± 6.5 in the web-based group (mean difference −1.7 ± 1.05 standard error), with similar results in the sensitivity test per modified intention-to-treat (Fig. 2). The lower limit of the one-sided 95% confidence interval (CI) did not exceed the predefined non-inferiority limit of *δ* = 5 points in both analyses (per protocol: one-sided 95% CI = −3.45; modified intention to treat: one-sided 95% CI = −1.95; Fig. 3). The participation in the web-based group was 14% higher. Incorrect adrenaline application and incorrect ventilation fraction, for example, were identified as recurring errors.

**Conclusion:**

Although underpowered to make a firm inference, our results suggest that web-based refresher is non-inferior compared to simulation-based refresher training, even in practical skills and shows a higher participation rate.

## Introduction

Newborn life support (NLS) is uncommon, with 0.1% of newborns requiring chest compressions, but always time-critical.^1^, ^2^, ^3^ There is evidence for a reduction in morbidity and mortality with NLS-training.^4^, ^5^, ^6^ Midwives, physicians and nurses must be well trained. The ideal time interval for training in NLS is unclear, but loss of knowledge and especially skills begins two to six months after training.^7^, ^8^, ^9^, ^10^ Therefore, refresher training seems rational after three months.^11^, ^12^ Training once a year is the minimum standard.^1^, ^2^, ^3^

Simulation-based training is effective and established.^4^, ^6^, ^13^, ^14^, ^15^, ^16^, ^17^ A number of studies have investigated various training modalities; however, these have been conducted on small, partly non-randomized groups, with unequal learning conditions and not in the context of multiprofessional teams.^18^, ^19^, ^20^, ^21^, ^22^, ^23^, ^24^ Regular team-oriented simulations are hampered by staff shortages, costs and non-availability of local courses.^12^

This multicenter randomized controlled trial (RCT) tested the non-inferiority of a web-based refresher compared to a simulation-based refresher course three months after a full-scale, high-fidelity simulation training. Knowledge and skills were assessed prior to and immediately after the initial training and six months later, i.e. three months after the refresher.

## Methods

### Ethical approval and consent

The master protocol of the RCT was approved by the Ethics Committee of the Medical Faculty of the Martin Luther University Halle-Wittenberg (22.08.2022, 2022-075) and registered with the German Clinical Trials Register on April 25, 2023 (https://www.drks.de/search/de/trial/DRKS00029152). The dataset, the statistical analysis code and the teaching materials are not publicly available due to privacy restrictions but can be partly obtained from the corresponding author upon reasonable request. All procedures were in accordance with laws and institutional guidelines. Written informed consent, including permission for video recording, was obtained before randomisation. A neonatologist who is also a pediatric advanced life support-instructor of the American Heart Association and responsible for NLS in-house training developed both training programs, supported by another physician and experts of an Education Center experienced in creating online learning materials and in Objective Structured Clinical Examination (OSCE).

### Sites and participants

The RCT was conducted in six hospitals Level I to IV in Germany. The five hospitals in rural areas, with different hospital operators, transfer critically ill neonates to the university hospital. The simulation-based training took place during working hours and with three to, with a few exceptions, a maximum of five healthcare professionals. We trained three healthcare professionals per group, if possible: one midwife, one nurse and one physician, as usually involved in a real NLS-situation. Inclusion criteria were certified healthcare professionals who are responsible for NLS and did not plan to terminate their employment before the last evaluation.

### Randomisation

On the day of written consent, healthcare professionals were randomized using random.org into two refresher groups (web-based vs. simulation-based), stratified for professional role (midwife, nurse and physician) in a 1:1 allocation ratio, regardless of age and sex. The stratification was necessary to ensure that medical and nursing staff remained evenly distributed, creating a realistic training situation.

### Study procedure

Before the first training, all participants received access to an interactive e-learning module based on the ERC NLS guideline.^25^ The full-scale high-fidelity simulation training in small interprofessional teams was delivered as a two-hour structured in-house training.

Each course included skills training and two full-scale simulations. These were standardized in terms of procedure, sequence of actions, trainer's reactions to participants' actions and duration. The first scenario was a simulation of meconium aspiration in a term neonate with respiratory failure and cardiac arrest (15 min). The simulation setting only differed from the working environment in monitoring, video documentation and the simulator. This scenario was for evaluation of baseline practical skills. Video documentation was used for debriefing and perception as well as discussion of the core content of Crew Resource Management (20 min). The subsequent skill training included learning objectives such as effective mask ventilation, recognition of chest elevation, MR SOPA mnemonic to improve ventilation, chest compressions, regular evaluation of heart rate without unnecessary interruption of ventilation, umbilical vein and intraosseous cannulation, adrenaline administration and advanced airway management.^26^, ^27^ Furthermore, leadership and communication skills were addressed (45 min).^28^, ^29^ In the second scenario a simulation with premature placental abruption, hypovolemic shock and cardiopulmonary arrest was performed (15 min), followed by debriefing (25 min). This schedule could be achieved by the ratio of instructors to participants (2:3).

Both refresher trainings consisted of repetitions of all core competences and were planned for 90 min.

Three months after the initial training, the intervention group (web-based group) received access to a custom-developed web-based refresher module consisting of eight compulsory and two optional units. To teach technical and non-technical skills we used simulation videos, real life videos and PowerPoint® presentations, altogether lasting 90 min (Table 1). This module was available for two months, latest 1 month before the assessment (questionnaire 3 and Megacode 3) and could be revisited multiple times. We did not record the frequency of access.

**Table 1:** Web-based material.

**Number**	**Content**	**Minutes**
1	Presentation of the most important aspects of the NLS algorithm with practical advice (11 slides)	11
2	Presentation of aspects of crisis resource management, including communication aids (10 slides)	10
3	A simulation scenario of a full-term infant with umbilical cord entanglement and cardiac arrest (including equipment check, management of ventilation problems, adrenaline administration via umbilical vein and intubation)	14
4	A simulation scenario of a preterm newborn at 32 weeks of gestation with asphyxia due to premature placental abruption (including resuscitation, intubation, adrenaline administration and emergency transfusion via umbilical vein)	10
5	A simulation scenario of a preterm newborn at 34 weeks of gestation with respiratory failure and the management of a pneumothorax	8
6	A skills training video about access routes via the umbilical vein	6
7	A skill training video about intraosseous access	4
8	A skills training video about bag-valve-mask ventilation, MR SOPA and nasopharyngeal intubation	6
9	A video about equipment-check and check of resuscitation environment	3
10	A real-life scenario of the initial care of a premature baby 26 weeks of gestational age with non-invasive respiratory support and less invasive surfactant application (with the consent of the parents)	13

The control group was invited to another simulation training at our local simulation center, with analogous content and time schedule to the web-based refresher and condensed in time regarding to the first training. The assessment took place three months after the refresher, by which time knowledge and skills may have partly deteriorated.

Data was collected from April 2023 to April 2024.

### Study outcome

The prespecified primary objective was to investigate the non-inferiority of a web-based refresher compared to a simulation-based refresher course in NLS. The overall assessment (0–60 points) consisted of a pseudonymised knowledge test (0–20 points), based on the questionnaire by Mileder and Gressl and the Megacode based on the validated Performance Checklist to Assess Neonatal Resuscitation Megacode Skill for practical skills (0–40 points) by Lockyer.^30^, ^31^ The first questionnaire also included socio-demographic questions and questions on working environment, work experience, self-efficacy, the latter measured by a three point Likert scale. In the light of the ERC 2021 recommendations, the questions were reviewed for relevance. We replaced two questions which focused on out-of-hospital delivery by two questions relevant for in-hospital deliveries (supplemental material: Questionnaire 1, question 15 and 17). The answer options in one question were deemed ambiguous by three neonatologists, we specified ‘medication’ by ‘adrenaline’ (supplemental material: Questionnaire 1, question 34). The Megacode by Lockyer was judged as suitable as it was a reliable and validated assessment tool at the time, all critical steps in NLS are included and corresponded to our scenarios as well as to the NLS-guideline, with three minor variations. We included in our assessment at item 1.7 that tone description was requested, in 1.9 the five initial ventilations for two to three seconds before further ventilation and the supply of oxygen if chest compressions were performed at item 1.15. In order to assess healthcare professionals who did not perform assisted ventilation or chest compressions during the simulation, they were asked to demonstrate these skills at the end of the scenario. Some items were addressed only once in the simulation, as is likely in real life, such as the need for adrenaline and volume. The contribution of each person to these shared-decision making items was evaluated.

On the day of the initial training, data was collected using questionnaire 1 and, in the initial scenario, using Megacode 1 before the actual teaching and immediately after the training using questionnaire 2, and in another scenario using Megacode 2. Three months after the refresher course the measurement was repeated using questionnaire 3 and using Megacode 3. As a priori secondary outcome, we compared attendance rates for both teaching methods. Recurring errors were identified directly during simulation and from review of the video.

### Sample size calculation and statistical analysis

The sample size calculation was based on a non-inferiority study design: web-based refresher is not inferior to full-scale simulation refresher training after initial simulation training of both groups in terms of knowledge and skill loss (H0: μweb-based > μsimulation-based − δ). The expected mean total score 3 months after the refresher was assumed to be 3 points higher in the simulation-based than in the web-based group, with a standard deviation of 5, with a *δ* = 5 points non-inferiority threshold.^32^ This resulted in a required number of 78 subjects per group with a significance level of *⍺* = 5% one-sided and a planned power of 80% (PASS software). With an expected 30% drop-out rate, derived from the study by Mosley (36% between two time points), 111 subjects per group were included.^3^, ^4^, ^7^, ^8^ Noninferiority was determined using a one-sided 95% confidence interval and considered proven if completely above the non-inferiority threshold (mean value of training on the simulation model minus *δ* = 5 points).

Multiple imputation was performed using the Markov chain Monte Carlo procedure for missing data.^33^, ^34^, ^35^ Secondary metric variables are described by mean, median, standard deviation and range; secondary categorical and dichotomous variables by absolute and relative frequencies. Metric data were compared by unpaired *t*-test and categorical variables by Chi square test. A *p*-value < 0.05 was considered as statistically significant. All analysis were conducted using SPSS® (IBM SPSS Statistics for Windows, Version 28.0 Armonk, NY, USA: IBM Corp).

## Results

**Fig. 1:**
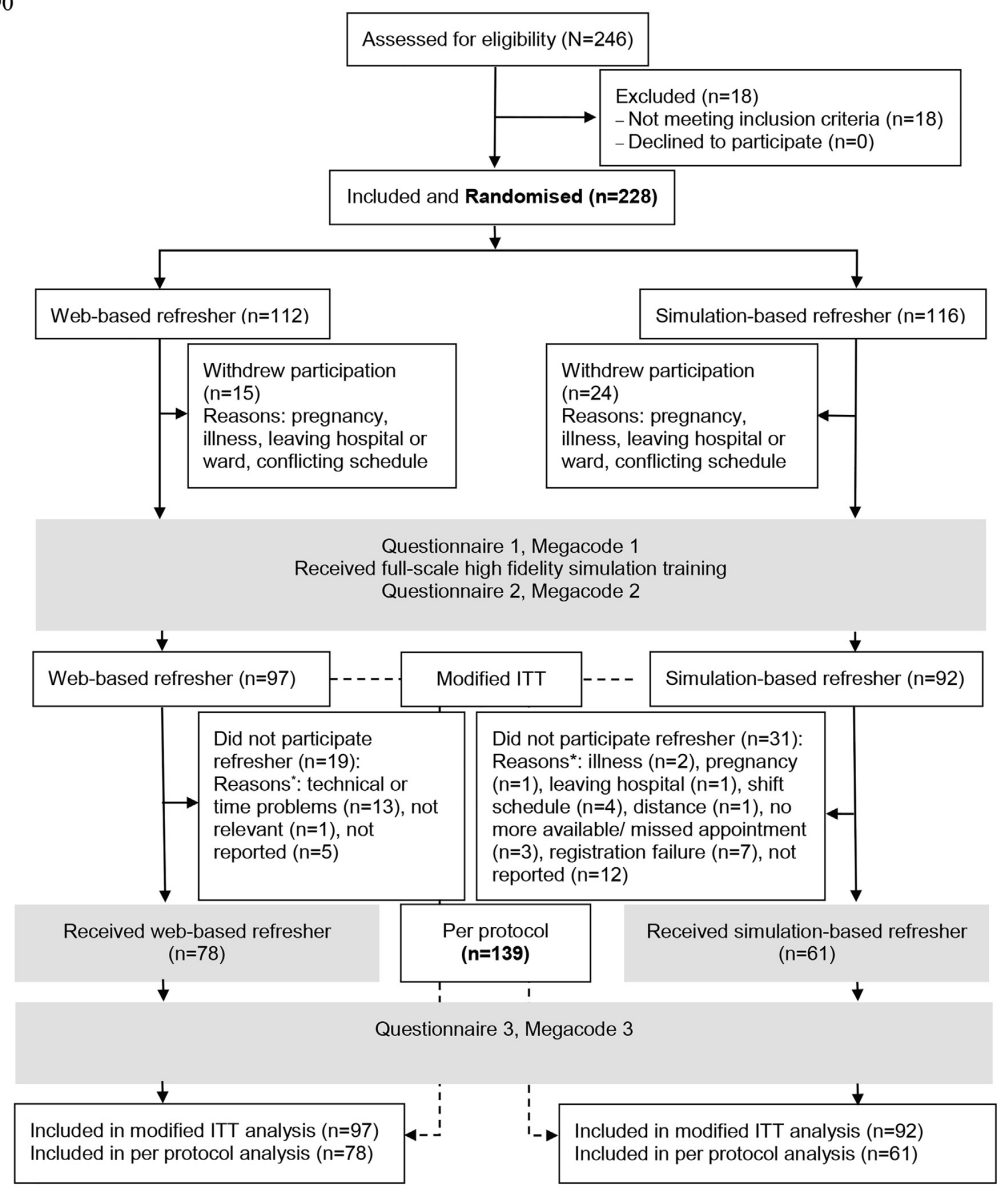
Flowchart. ITT = intention-to-treat, *Reasons according to returned evaluation forms (83 in total, 40 web-based and 43 simulation-based).

246 of 282 healthcare professionals responsible for NLS were assessed for eligibility. 228 healthcare professionals meeting the inclusion criteria consented to participation, and were randomized. 39 of these withdrew prior to the questionnaire 1 and Megacode 1 (Fig. 1). To rule out systematic dropout, differences between participants and non-participants were examined with no relevant difference regarding age, gender and profession (Table 2S). Baseline characteristics, work-experience and self-efficacy of participants are shown in Table 2. Comparison between both refresher groups showed similar baseline knowledge and skills, with equal improvement from the first to the second assessment (Table 3).

**Table 2:** Baseline characteristics, including sociodemographic data, work-experience and self-efficacy.

**Variable**	**Web-based** ***n* = 97**	**Simulation-based** ***n* = 92**	**Overall** ***N* = 189**
Age, years	41.1 (13.1, 20–63)	39.2 (11.4, 22–61)	40.2 (12.3, 20–63)
**Sex**			
Female	81 (83.5)	78 (84.8)	159 (84.1)
Male	16 (16.5)	14 (15.2)	30 (15.9)
**Profession**			
Physician	42 (43.3)	43 (46.7)	85 (45.0)
Pediatrician	24 (24.7)	25 (27.2)	49 (25.9)
Obstetrician	12 (12.4)	14 (15.2)	26 (13.8)
Anesthetist	6 (6.2)	4 (4.3)	10 (5.3)
Nurse	31 (31.9)	29 (31.5)	60 (31.7)
Anesthetic	4 (4.1)	2 (2.2)	6 (3.2)
Pediatric	27 (27.8)	25 (27.1)	52 (27.5)
No specialization	0 (0)	2 (2.2)	2 (1.1)
Midwife	24 (24.7)	20 (21.7)	44 (23.3)
**Experience at a neonatal ward**†			
No experience	47 (48.5)	41 (44.6)	88 (46.6)
Less than 6 months	11 (11.3)	17 (18.5)	28 (14.8)
6 months–1 year	5 (5.2)	6 (6.5)	11 (5.8)
More than 1 year	29 (29.9)	26 (28.3)	55 (29.0)
**Never seen or used**			
Assisted ventilation	20 (20.6)	23 (25.0)	43 (22.8)
Invasive ventilation	40 (41.2)	42 (45.7)	82 (43.4)
Chest- compression	42 (43.3)	55 (59.8)	97 (51.3)
Use of adrenaline	56 (57.7)	65 (70.7)	121 (64.0)
Trained in NLS*	40 (41.2)	45 (48.9)	85 (45.0)
Instructor, unspecified	1 (1.0)	1 (1.1)	2 (1.1)
Frequency of high self-efficacy in NLS*, †			
In general/most often	26 (26.8)	26 (28.3)	52 (46.0)
Frequently (50%)	32 (33.0)	21 (22.8)	53 (28.0)
Rarely/Never	28 (28.9)	33 (35.9)	61 (32.3)
**Wish for support of self-efficacy**			
Theory-Repetition	67 (69.1)	65 (70.7)	132 (69.8)
Simulation-Repetition	85 (87.6)	88 (95.7)	173 (91.5)
Observing NLS	43 (44.3)	45 (48.9)	88 (46.6)

Data are mean ± standard deviation or No. (%) and range for age.

* NLS, Newborn Life Support.

† If percentages do not add to 100%, data was missing in the questionnaire.

**Table 3:** Competence score for knowledge and skills (per protocol und modified intention to treat).

**Competence Score** **Mean (SD)**	**Per protocol**			**Modified intention to treat**		
	**Web-based participants** ***n* = 78**	**Simulation-based participants** ***n* = 61**	***p***	**Web-based participants** ***n* = 97**	**Simulation-based participants** ***n* = 92**	***p***
**Theoretical Knowledge Score** **Questionnaire**						
1	13.1 (±2.7)	14.3 (±2.4)	0.01	13.0 (±2.8)	13.8 (±2.7)	0.53
2	16.5 (±2.1)	16.7 (±2.5)	0.77	16.4 (±2.2)	16.5 (±2.7)	0.74
3	15.7 (±1.9)	15.7 (±2.0)	0.90	15.4 (±2.2)	15.2 (±2.7)	0.53

**Practical Skills Score** **Megacode**						
1	21.9 (±7.5)	21.8 (±7.9)	0.99	21.1 (±7.6)	21.3 (±8.0)	0.87
2	31.5 (±6.2)	32.8 (±4.8)	0.17	31.2 (±6.2)	32.4 (±4.7)	0.14
3	29.7 (±5.6)	31.3 (±5.0)	0.08	29.7 (±5.5)	30.0 (±5.6)	0.65

Data are mean ± standard deviation and significance.

### Primary outcome

**Fig. 2:**
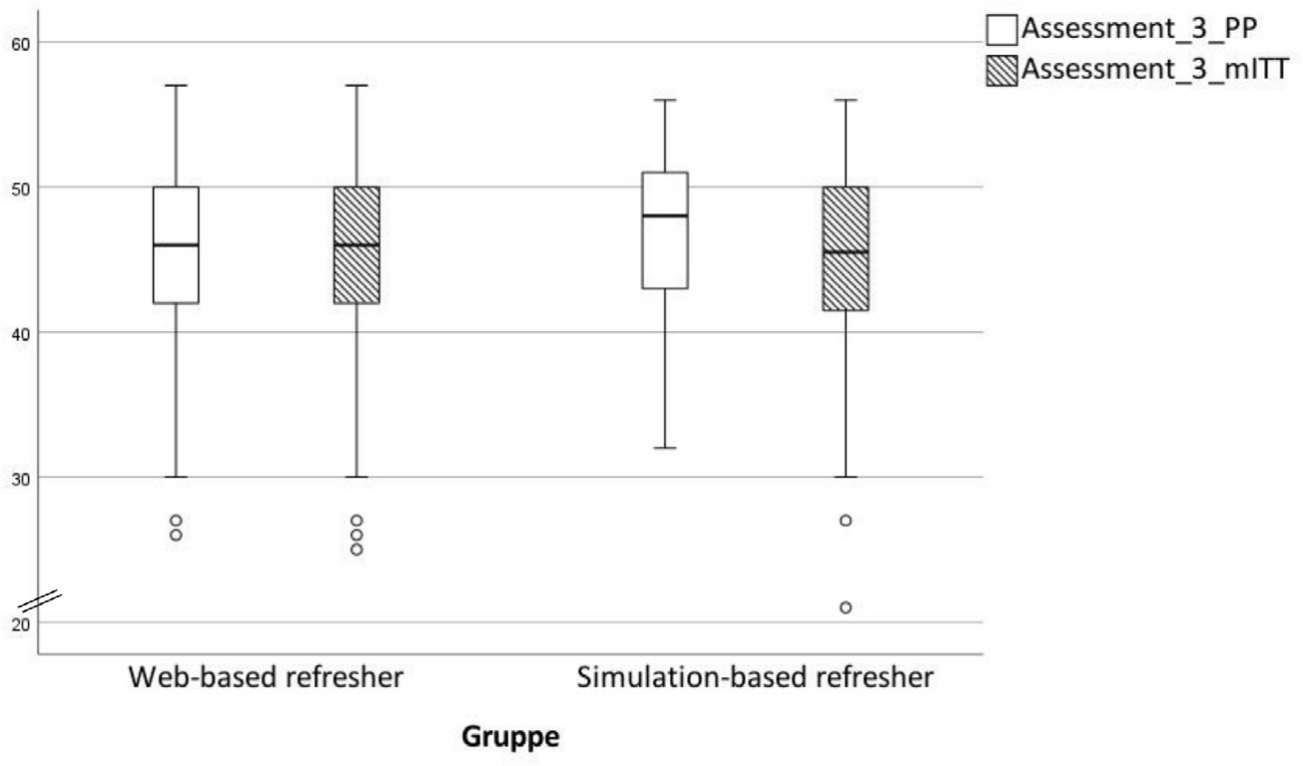
**Boxplots comparing the total score of the assessment (0–60 points) 3 months after the refresher, consisting of knowledge (0–20 points) and practical skills (0–40 points) in the web-based refresher group with the simulation-based refresher group in the per protocol (pp; *n* = 139) and modified intention-to-treat (mITT; *n* = 189) analysis set**.

**Fig. 3:**
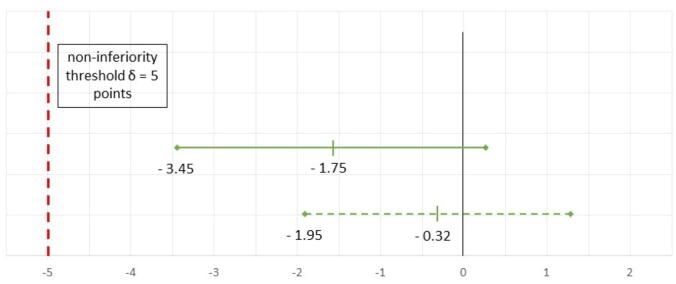
**Non-inferiority testing using per protocol and modified intention-to-treat analysis. The lower limit of the one-sided 95% confidence interval (CI) does not exceed the predefined non-inferiority limit of *δ* = 5 points in either the per protocol analysis (mean difference = −1.75, lower CI limit = −3.45; solid green line) nor the modified intention to treat (mean difference = −0.32, lower CI limit = −1.95, dashed green line); *n* = 139 (per protocol); *n* = 189 (modified intention to treat)**. (For interpretation of the references to color in this figure legend, the reader is referred to the web version of this article.)

The mean ± SD sum scores from theory and practice 3 months after the refresher (questionnaire 3 + Megacode 3) in the per protocol analysis were 45.2 ± 6.5 in the web-based and 47.0 ± 5.6 in the simulation-based group (mean difference −1.75 ± 1.05 SE). In the sensitivity test per modified intention to treat, the mean ± SD sum scores were 44.9 ± 6.6 in the web-based and 45.2 ± 6.9 in the simulation-based group (mean difference −0.32 ± 0.98 SE; Fig. 2). The lower limit of the one-sided 95% CI did not exceed the predefined non-inferiority limit of *δ* = 5 points in both analyses (per protocol: one-sided 95% CI = −3.45; modified intention to treat: one-sided 95% CI = −1.95; Fig. 3).

### Boxplots comparing the total score of the assessment

#### A priori secondary outcomes

Participation in the web-based refresher was higher than in the simulation-based refresher (80% (78/97) vs. 66% (61/92)). 70% (68/97) of participants in the web-based and 72% (66/92) in the simulation-based group would like to repeat the simulation within 3–6 months at the latest.

With Megacode 3, not stratified by randomization group we observed 74 teams with failure in re-evaluations intervals in 81% (60/74) and interruptions of ventilation during reassessment in 45% (33/74). Further a lack of flushing the cannula before installation (36/74, 49%) and after adrenaline (28/74, 38%) and overly-long intubation-attempts were observed. No significant differences were found between the web-based and simulation-based groups 3 months after the refresher regarding reassessment on time (25% vs 14%; *p* = 0.19), continued ventilation during handover and re-evaluation (78% vs 77%; *p* = 0.91 and 53% vs 51%; *p* = 0.90) and flushing the cannula before insertion and after adrenaline administration (54% vs 53%; *p* = 0.90 and 61% vs 62%; *p* = 0.89). Other recurring errors were observed regarding temperature management, review of chest elevation, call for help, chest compression, intubation- and communication-skills without further differentiation.

## Discussion

Following an initial full-scale, high-fidelity simulation training we compared a web-based and a simulation-based refresher in newborn life support. Our data suggest that the web-based refresher was non-inferior to the simulation-based refresher, even in practical skills. However, the required sample size of 78 subjects in the simulation-based refresher group could not be achieved for per protocol analysis to conclusively answer this question. The scores show comparable results in the simulation-based compared to the web-based group in the modified intention to treat analysis. 31 participants of the simulation-based versus 19 of the web-based group were not refreshed and therefore the knowledge and skills might be a result from the first training and relation to the refresher is unclear. A third group without intervention would be the best option to clarify this question.

It is important to consider a variety of reasons for the fact that some healthcare professionals were not available for refresher training. Chief among them are the scheduling issues that have been highlighted. However, it is important to note that the period of three months may not be considered effective or necessary by professionals. The assessment for knowledge and skill retention was 3 months after the refresher, as a reasonable and achievable time for spaced repetition, since some studies showed loss of competences, starting two to six months after training.

The possibility of repeatedly access to the online material for the web-based group over a period of two month until four weeks before the final assessment might have influenced the results in favor of the web-based group. The simulation-based group had only one 90-min refresher course three months before the assessment. But since unrestricted use is one advantage of online material, we deliberately enabled the web-based group to use the material as often as desired for two months. Tracking frequency of access would provide further information.

As randomisation and similar in-house teaching at the participants’ hospitals suggest that there were no group differences prior to the refresher training, no assessment was undertaken beforehand or after the refresher, since this would need another simulation for the web-based participants. That would influence the results. And our aim was to investigate the retention of knowledge and skills at a time point, where we think a refresher is reasonable.

Only few previous analyses have used a design comparable to our study (initial full-scale simulation training with further on-site or web-based training). In an analysis, nursing students were sent videos of their most recent training session, starting three months after an advanced life support simulation training course.^36^ Six months after the initial training these students, in contrast to those without a refresher, showed significantly better results in terms of skills and self-efficacy. The loss of knowledge was less in the intervention group, but the difference was not statistically significant. Another study found no differences between nursing students trained in person and those trained by video.^8^ In contrast to our study a simulation format was used to analyze a single skill. Gaming might be an alternative, but studies show conflicting results.^20^, ^23^, ^37^, ^38^

In our study, nearly 10% (3/31) of participants in the simulation-based refresher group cited shift schedules as a reason for non-participation. The higher participation in the web-based refresher, might be explained by the reduced time and travel expenditure, as well as less schedule restrictions.^12^ Regular training should be included in working hours. In-house training showed a higher participation rate than traveling to a simulation center, since all healthcare professionals of the initial training could be trained again 6 months later in-house. However, a refresher every three months might not be considered necessary and therefore explain the non-participation, but not the difference in the both groups. Web-based refresher might help to increase booster participation.

We showed that even for experienced healthcare professionals, the training must cover all the NLS-basics and all items of the Megacode should be covered in the refresher course (Table 2, Table 3).

The Megacode by Lockyer et al. (adapted in three items regarding the 2021 guideline) was used even though it was developed for the Neonatal Resuscitation Program 2005, since there was no more suitable Megacode to our simulation at the start of our study.^25^, ^30^ Unlike the new Megacode, however, we would recommend covering all core procedures including administration of medication.^39^ Fluid administration is essential in any bleeding conditions like placental abruption and adrenaline was not always administered correctly both in terms of dosage as well as flushing into the vein in both groups. The adrenalin administration reached levels up to ten times the recommended dose.

Re-evaluation every 30 s is important and based on physiology, but more than 80% of the teams did not perform reassessment in time, regardless of group. Suboptimal heart rate assessment and airway management as well as too short chest compression time is reported in the literature.^40^, ^41^ A new Megacode includes the heart rate assessment every 30 s.^39^ Technical solutions might be useful to optimize the processes. Interruptions of ventilation and overly-long intubation attempts were often seen in our as well as other studies and represent a reason for unsuccessful or prolonged resuscitation.^42^, ^43^, ^44^, ^45^

In NLS, the team, comprising members from various professional groups, is crucial.^46^, ^47^, ^48^ Therefore NLS training should be conducted within the team and all formats should address all healthcare professionals responsible for newborns, best performed in multiprofessional simulation trainings. In addition, all healthcare professionals who may be involved in NLS should be well trained according to defined learning objectives, ideally more than once a year. Web-based formats support personalized learning, whilst simulations enhance team performance, communication and technical skills.

Our results suggest the noninferiority of a web-based compared to a simulation-based refresher at a three-monthly interval, however limited by the underpowered study. The study has demonstrated that with an in-house training every six months we reached most of the healthcare professionals. However, a higher proportion of professionals participate in their allocated refresher training when it is web-based rather than traveling to an external simulation center in a planned refresher three month after a full-scale simulation training.

### Limitations

This project has important limitations. Participation was voluntary. Therefore, the results may have been influenced by the higher knowledge and skills of those interested. We cannot rule out that the assessment-score 3 was influenced by the initial training. A third group without refresher training would have answered this question. Future studies should examine whether knowledge loss begins approximately six months rather than three months after comprehensive simulation training in multiprofessional teams.

Additionally, it remains unclear whether this intervention had impact on neonatal mortality and morbidity.

**TRIAL REGISTRATION German Clinical Trials Register (DRKS) 00029152,** 25.04.2023, https://drks.de/search/en/trial/DRKS00029152.

## CRediT authorship contribution statement

**Kathleen Parthey:** Writing – review & editing, Writing – original draft, Visualization, Validation, Resources, Project administration, Methodology, Investigation, Funding acquisition, Formal analysis, Data curation, Conceptualization. **Anne Goldhahn:** Writing – review & editing, Resources, Project administration, Investigation, Data curation. **Anselm Linke:** Writing – review & editing, Resources, Investigation, Data curation. **Katja Raberger:** Writing – review & editing, Supervision, Funding acquisition. **A. Wienke:** Writing – review & editing, Validation, Methodology, Formal analysis. **Dietrich Stoevesandt:** Writing – review & editing, Supervision, Resources. **Roland Haase:** Writing – review & editing, Supervision, Resources, Funding acquisition.

## Funding

Donations worth 5.000€ were raised for the study. There were no financial relationships with the participating hospitals or those involved in the study, but the donor, a local transport company, does not wish to be named in the publication. The information has been shared. A research assistant, training materials and part of the travel costs were financed by the University Medical Center Halle of funding for young scientists with educational and nursing responsibilities, the remaining travel costs were covered by the study doctors themselves with 2801,20 €. The funders had no influence on the design, conduct, analysis and reporting of the trial.

## Declaration of competing interest

The corresponding author K.P. reported no conflict of interest but receiving personal fees, for giving lectures as an AHA Instructor for PALS courses outside the submitted work.

None of the co-authors have any conflicts of interest to declare. There are no financial conflicts of interest to resolve.

## Appendix A Supplementary material

Supplementary Data 1 Comparision to drop-out professionels and Questionnaire 1.
