# Supplementary material for: Web-based versus simulation-based refresher training in newborn life support: A randomized multicenter noninferiority controlled trial
Source: Resusc Plus. 2026 Jun 10;30:101382. doi: 10.1016/j.resplu.2026.101382 (PMC13310597; doi:10.1016/j.resplu.2026.101382)
Supplement: Supplementary Data 1 — Comparision to drop-out professionels and Questionnaire 1. [file mmc1.docx]

#### Supplement

Table 1S. Comparision of Baseline-Characteristics in mITT and drop out population

|  | mITT  web-based  n= 97 | Drop out  web-based  n= 15 | p | mITT  simulation-based  n= 92 | Drop out  simulation-based  n= 23 | p |
| --- | --- | --- | --- | --- | --- | --- |
| **Age, mean, year** | 41.06  (13.05) | 42.00 (11.22) | 0.79 | 39.21  (11.43) | 35.75 (12.66) | 0.20 |
| **Sex**  Female  Male | 81  16 | 13  2 | 0.76 | 78  14 | 21  3 | 0.74 |
| **Profession** |  |  |  |  |  |  |
| Physician | 42 | 7 | 1.0 | 43 | 7 | 0.17 |
| Nursing staff | 31 | 1 | 0.06 | 29 | 10 | 0.47 |
| Midwife | 24 | 7 | 0.12 | 20 | 7 | 0.43 |

Data are mean ± standard deviation or No. (%). p < 0.05

#### Questionnaire 1

#### (Translation to English with ChatGPT based on Modell GPT-5.3 by OpenAI)

Pseudonym: _____________________ Date: __________

#### General questions

**1. Profession:**

**Consultant**

☐ Anaesthesia ☐ Obstetrics & Gynaecology ☐ Pediatrics

**Registrar**

☐ Anaesthesia ☐ Obstetrics & Gynaecology ☐ Pediatrics

**Specialist**

☐ Anaesthesia ☐ Obstetrics & Gynaecology ☐ Pediatrics

**Junior doctor (>2 years’ experience)**

☐ Anaesthesia ☐ Obstetrics & Gynaecology ☐ Pediatrics

**Junior doctor (<2 years’ experience)**

☐ Anaesthesia ☐ Obstetrics & Gynaecology ☐ Pediatrics

**Nurse**

☐ Anaesthetic nurse (>2 years’ experience) ☐ Anaesthetic nurse (<2 years’ experience)

☐ Paediatric nurse (>2 years’ experience) ☐ Paediatric nurse (<2 years’ experience)

**Midwife**

☐ Midwife (>2 years’ experience) ☐ Midwife (<2 years’ experience)

2. Have you worked on a neonatal unit in the last 5 years?

☐ No ☐ Yes – if yes, please indicate duration:

☐ 6 months ☐ 1–2 years ☐ 2–3 years ☐ >3 years

3. Have you performed ventilation/resuscitation in the delivery room within the last 5 years?

☐ Yes ☐ No

If yes:

3.1 Use of respiratory support?

☐ 0 times ☐ 1 time ☐ 2–3 times ☐ 4–6 times ☐ >6 times

3.2 Use of invasive ventilation?

☐ 0 times ☐ 1 time ☐ 2–3 times ☐ 4–6 times ☐ >6 times

3.3 Use of chest compressions?

☐ 0 times ☐ 1 time ☐ 2–3 times ☐ 4–6 times ☐ >6 times

3.4 Use of adrenaline?

☐ 0 times ☐ 1 time ☐ 2–3 times ☐ 4–6 times ☐ >6 times

3.5 When were you last part of a resuscitation/ventilation team in the delivery room?

☐ Within the last year ☐ 1–2 years ☐ 2–3 years ☐ >3 years

4. Have you attended an NLS training/newborn emergency course?

☐ Yes, when: __________________ where: __________________

☐ No

5. Are you certified as a PALS or NLS instructor?

☐ Yes, when: __________________

☐ No

6. Do you feel efficient/confident/prepared for neonatal resuscitation?

☐ Never to rarely ☐ Sometimes ☐ Often to always

7. Do you conduct a briefing in the delivery room?

☐ I am not familiar with this

☐ Never ☐ Rarely ☐ Sometimes ☐ Often ☐ Always

8. Is debriefing conducted in your hospital?

☐ I am not familiar with this

☐ Never ☐ Rarely ☐ Sometimes ☐ Often ☐ Always

9. What could improve your confidence in neonatal resuscitation?

☐ Nothing

☐ Repetition of theoretical training every ______ months

☐ Repetition of NLS simulation every ______ months

☐ Observing resuscitation scenarios

☐ Access to telemedical support

10. Who do you call in an emergency? Telephone number:

________________________________________________

11. Are you familiar with the equipment in the delivery room?

☐ Yes ☐ No

12. Where are the ventilation masks located?

________________________________________________

13. Where are the suction catheters located?

________________________________________________

14. Can you check and operate the ventilator?

☐ Yes ☐ No

**Single best answer questions:**

15. During chest compressions, FiO2 is always set to 1.0 (100%).

☐ True ☐ False

16. What should be ensured when preparing to manage a critically ill newborn?

☐ Warm, well-lit, draught-free environment

☐ Well-lit environment with open windows for fresh air

☐ Warm, well-lit environment with open windows

☐ Fresh air and, if possible, therapeutic hypothermia

17. What does the acronym MR SOPA stand for?

☐ Mask adjustment, Repositioning of head/airway, Suctioning, Opening the mouth, Pressure increase, Alternative airway

☐ Equipment preparation sequence

☐ Clinical checklist

18. When should uncomplicated, newborns be clamped?

☐ Immediately ☐ ≥1 min ☐ ≥3 min ☐ ≥5 min

19. Recommended room temperature for the initial care of newborns?

☐ 20–23°C ☐ 23–25°C ☐ 25–28°C ☐ >28°C

20. What are the recommended initial assessment parameters?

☐ Breathing, heart rate (HR), reflexes

☐ Breathing, heart rate (HR), muscle tone

☐ Breathing, heart rate (HR), colour

☐ Breathing, colour, muscle tone

21. What do newborns with inadequate breathing and heart rate <100 usually require?

☐ Drying only

☐ Drying and mask ventilation

☐ Ventilation and chest compressions

☐ Medication

22. What are the first steps in apnoeic newborns?

☐ Start chest compressions

☐ Head tilt and ventilation

☐ Open airway and provide 5 initial breaths

☐ Immediate intubation

23. When should suction be performed?

☐ Always

☐ Only if airway obstruction is present

☐ Only nasopharyngeal suction

☐ Only during cardiopulmonary resuscitation

24. How should initial breaths be delivered?

☐ Sustained inflation pressure for 2–3 seconds

☐ Increasing pressure during breaths

☐ Expiratory pressure held constant

☐ Using full bag volume

25. What should you do if there is no chest rise during ventilation?

☐ Intubation

☐ Auscultation to exclude pneumothorax

☐ Reposition airway and repeat initial breaths

☐ Repeat breaths only

26. What is the initial oxygen concentration for term infants?

☐ 0% ☐ 21% ☐ 79% ☐ 100%

27. By when should SpO2 reach 90% in term neonates?

☐ 1 min ☐ 3 min ☐ 5 min ☐ 10 min

28. What is required for effective chest compressions?

☐ Adequate room temperature

☐ Heart rate <100

☐ Effective ventilation established beforehand

☐ Defibrillator available

29. When should chest compressions be started?

☐ Always

☐ No detectable heart rate

☐ Heart rate <60 despite effective ventilation

☐ If ventilation is ineffective

30. What is the correct ratio of chest compressions to ventilations?

☐ 3:1 ☐ 5:2 ☐ 15:2 ☐ 30:2

31. What is the correct chest compression rate per minute?

☐ 90 ☐ 100 ☐ 120 ☐ 160

32. What is the recommended compression depth and technique?

☐ Half of chest depth, two-finger technique

☐ One-third chest depth, two-thumb technique

☐ One-quarter chest depth, two-thumb technique

☐ One-third chest depth, two-finger technique

33. How often should heart rate be reassessed during resuscitation?

☐ Every 30 seconds ☐ Every 1 minute ☐ Every 2 minutes ☐ Not required

34. When should adrenaline be considered?

☐ Always within 30 seconds

☐ Heart rate <60 despite ventilation and chest compressions

☐ Heart rate <100 despite ventilation and chest compressions

☐ When a neonatologist arrives

35. Are you familiar with telemedical support?

☐ Yes ☐ No

Telemedical support allows remote neonatology consultation via video.

36. Would you like telemedical support in neonatal emergencies?

☐ Yes ☐ No ☐ Unsure

37. Would you like telemedical support for decisions to stop resuscitation?

☐ Yes ☐ No ☐ Unsure

38. Would you like telemedical support for severe congenital anomalies?

☐ Yes ☐ No ☐ Unsure

39. How often would you have liked telemedical support in the last 2 years?

☐ 0 ☐ 1 ☐ 2–3 ☐ 4–6 ☐ >6

40. In which situations would you have liked telemedical support?

________________________________________________

________________________________________________

________________________________________________
